# Confined environments induce polarized paraspeckle condensates

**DOI:** 10.1038/s42003-023-04528-4

**Published:** 2023-02-03

**Authors:** Vanja Todorovski, Finn McCluggage, Yixuan Li, Annika Meid, Joachim P. Spatz, Andrew W. Holle, Archa H. Fox, Yu Suk Choi

**Affiliations:** 1 School of Human Sciences, The University of Western Australia, Crawley, 6009 WA Australia; 2 Mechanobiology Institute, National University of Singapore, 117411 Singapore, Singapore; 3 Department of Cellular Biophysics, Max Planck Institute for Medical Research, Heidelberg, 69120 Germany; 4 Department of Biophysical Chemistry, University of Heidelberg, Heidelberg, 69117 Germany; 5 Department of Biomedical Engineering, National University of Singapore, 117411 Singapore, Singapore

**Keywords:** Biophysics, Long non-coding RNAs, Cell migration

## Abstract

Cancer cells experience confinement as they navigate the tumour microenvironment during metastasis. Recent studies have revealed that the nucleus can function as a ‘ruler’ for measuring physical confinement via membrane tension, allowing for compression-sensitive changes in migration. Cell nuclei contain many nuclear bodies that form when their components phase separate and condense within permissive local regions within the nucleus. However, how sub-nuclear organisation and phase separation changes with cell confinement and compression is largely unknown. Here we focus on paraspeckles, stress-responsive subnuclear bodies that form by phase separation around the long non-coding RNA NEAT1. As cells entered moderate confinement, a significant increase in paraspeckle number and size was observed compared to unconfined cells. Paraspeckle polarization bias towards the leading edge was also observed in confinement, correlating with regions of euchromatin. Increasing paraspeckle abundance resulted in increases in confined migration likelihood, speed, and directionality, as well as an enhancement of paraspeckle polarization towards the leading edge. This polarization of paraspeckle condensates may play a key role in regulating confined migration and invasion in cancer cells, and illustrates the utility of microchannel-based assays for identifying phenomena not observed on 2D or 3D bulk substrates.

## Abstract (web summary)

In a breast cancer cell line migrating through microchannels, stress-responsive paraspeckle numbers increase under confinement and polarize towards the leading edge.

## Introduction

As the largest and stiffest organelle, the nucleus is particularly susceptible to deformation in confinement. Accordingly, the nucleus can sense compression and mediate the cellular response to confinement smaller than the nuclear diameter^1^. Active strain, magnetic twisting cytometry, dynamic tensile loading, and geometric constraints have all been utilized to induce changes in chromatin state, subsequently leading to changes in global gene expression^2^. Such changes in gene expression are not only associated with changes in chromatin compaction but are also connected to alterations in the subnuclear organization, including the distribution and organization of nuclear bodies. Recent breakthroughs have explained how the formation of nuclear bodies and chromatin micro-environments are dependent on the phase separation of component molecules. However, the degree to which external mechanical forces influence the nuclear interior, and thereby phase separation, remains largely unknown. Deciphering the connections between mechanical forces and nuclear remodeling is important to aid the understanding of mechano-nuclear interactions during cancer metastasis. This is especially relevant in the context of recent findings on the nature of fluid-filled interstitial spaces in tissues that provide mechanical confinement and compression on migrating cells^3^. This confined migration includes cancer invasion through micron-sized tracks of collagen fibers and squeezing through submicron-sized endothelial junctions^4,5^.

Paraspeckles are stress-sensitive nuclear bodies formed via the transcription of the long non-coding RNA (lncRNA) NEAT1^6^ and subsequent liquid-liquid phase separation of proteins that bind the RNA. Recently, we have shown that paraspeckle formation in metastatic cancer cells is sensitive to cell culture substrate stiffness^7,8^ and others have shown a paraspeckle increase in osteoclasts subject to mechanical loading^9^. NEAT1 possesses two isoforms, 3.7 kb NEAT1_1 and 23 kb NEAT1_2, with the latter playing the dominant role in the paraspeckle formation. Transiently increasing paraspeckle abundance is possible via forced alteration of NEAT1 isoform ratios using targeted antisense oligonucleotides^10^. This paraspeckle upregulation mirrors natural stress-induced paraspeckle induction, which is linked to increased cell viability in the face of stress^6^. Functionally, paraspeckles act as gene transcriptional hubs that influence genome organization through RNA–RNA interactions^11^, as well as sequestering specific proteins and RNAs, altering transcriptional and post-transcriptional gene regulation^6^. Importantly, NEAT1 expression has been shown to correlate with HER2-positive breast cancers^12^ and play an essential role in metabolic changes that promote breast cancer growth and metastasis^13^, suggesting that paraspeckle dynamics may play a role in disease progression.

Given our own observations of stiffness-sensitive paraspeckle dynamics, as well as other recent work connecting paraspeckles and nuclear size, we speculated that paraspeckles may also be sensitive to confinement-induced nuclear compression^7,8^. As the nucleus is an active regulator of confined migration, we further examined whether transiently increasing paraspeckle abundance by altering NEAT1 levels would affect migration in confining microchannels^1^.

## Results

### Confinement induces major changes in paraspeckle formation and morphology

**Fig. 1:**
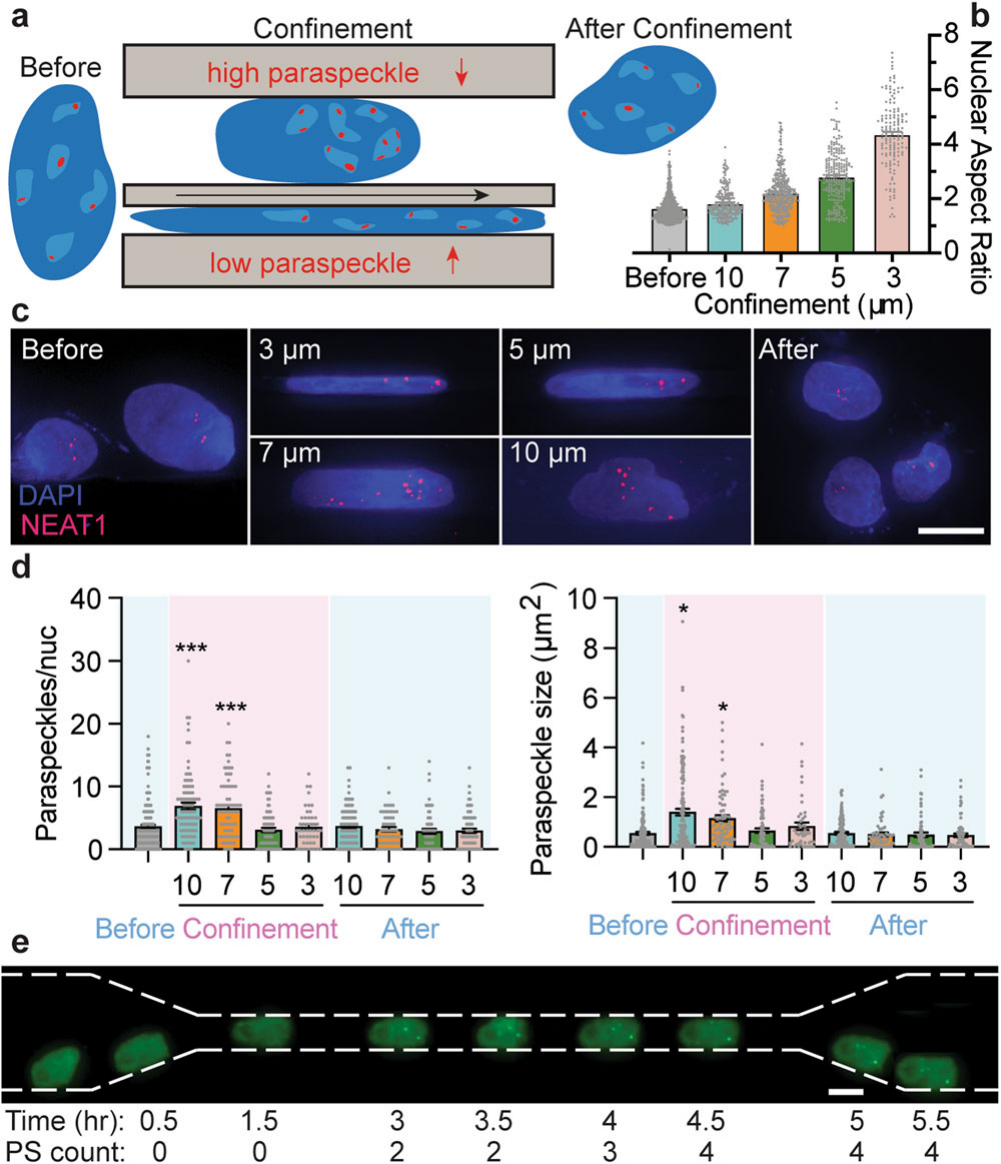
Paraspeckle formation is confinement-dependent. **a** Schematic illustrating the nuclei in the inner reservoir pre-confinement (before), in moderate (10 µm) and extreme (3 µm) confinement, and post-confinement. **b** Nuclear aspect ratio scales with confinement degree. All conditions are significantly different from each other. **c** Representative RNA-FISH images showing paraspeckle abundance (Scale bar, 10 µm). **d** Bar graphs show the average number of paraspeckles per nucleus (left) and the total area of individual paraspeckles (right). **e** Superimposed time-lapse video of a cell migrating through a 10 µm channel. All data are shown as mean ± SEM *P < 0.05 and ***P < 0.001. All scale bars = 10 µm.

Given the connection between NEAT1 expression and breast cancer dynamics, as well as their previously demonstrated ability to migrate in a wide variety of confining spaces^14^, we seeded MDA-MB-231 breast cancer cells in the inner reservoir of microchannel chips (Fig. 1a), resulting in differential levels of nuclear deformation during confined migration (Fig. 1b). After 72 h, cells were fixed and FISH against NEAT1 was performed to detect paraspeckles. NEAT1 FISH is a robust paraspeckle readout, giving precise co-localization with the paraspeckle marker protein SFPQ in fixed cells (Supplementary Fig. 1). While migration into extreme confinement (3 or 5 µm microchannels) did not change paraspeckle expression, cells within moderately confining microenvironments (7 and 10 µm microchannels) exhibited a nearly twofold increase in nuclear paraspeckles (Fig. 1c, d). This increase in nuclear paraspeckles was not due to global changes in NEAT1 levels as measured by FISH signal across the cell, which was found to be equivalent in confined and unconfined cells (Supplementary Fig. 2). This paraspeckle upregulation was not permanent, as cells that had migrated through the microchannels to the outer reservoir did not show a significant difference in paraspeckle frequency compared to those in the inner reservoir that were never exposed to confinement. Paraspeckle morphology was also influenced by confinement, with 10 µm confinement leading to a near tripling in the total paraspeckle area (Fig. 1d). This paraspeckle enhancement was observed dynamically in live cells expressing GFP-tagged SFPQ, with the appearance of new paraspeckles occurring within 45 min after cells entered the confinement (Fig. 1e, Supplementary Video 1). As paraspeckle dynamics are a balance between the formation and degradation of condensates, we also measured paraspeckle lifetime in 2D conditions to determine if the increase observed in confinement was due to a mechanical activation of phase separation or a mechanical reduction of degradation. When observed for a period of 10 h, less than 10% of paraspeckles were found to dissociate in less than three hours, the maximum time required for cells to permeate through the confining microchannel (Supplementary Fig. 3). This indicates that the nearly twofold increase in paraspeckle abundance in confinement is due to the enhanced formation, not enhanced stability.

### Paraspeckle boosting drives enhanced confined migration

**Fig. 2:**
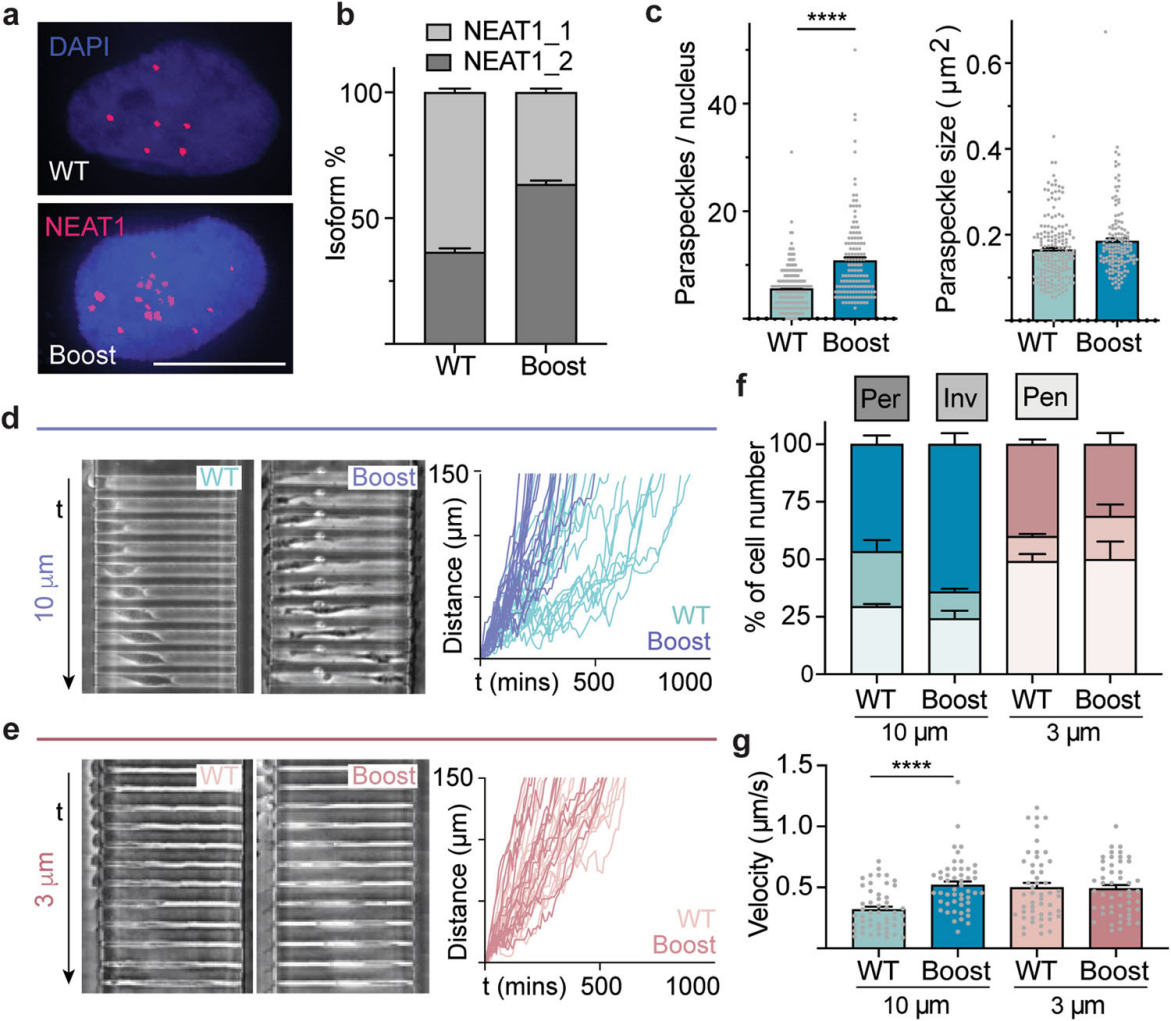
Boosting paraspeckle abundance enhances invasion into confinement. **a** RNA-FISH images of nuclei confirming increased paraspeckle abundance as a result of forced NEAT1_1-NEAT1_2 isoform switching. **b**, **c** Isoform switching resulted in an increase in the number of paraspeckles per nucleus (left) without significantly changing paraspeckle size (right). Isoform switching experiments were repeated 3 times and confirmed by qPCR. **d**, **e** Kymographs (left) and leading edge tracks (right) of control and boosted cells in moderate and extreme confinement. Phase contrast images comprising kymographs taken every 10 min. **f** Proportions of cells penetrating (Pen), invading (Inv), or permeating (Per) the microchannels as a function of confinement and paraspeckle abundance. 50 cells were analyzed per condition. **g** Cell velocity as a function of confinement and paraspeckle abundance. All data are shown as mean ± SEM ****P < 0.0001. All scale bars = 10 µm.

While confinement had a clear effect on paraspeckle dynamics, it was unclear if paraspeckle regulation could affect the ability of cells to undergo confined migration. To transiently increase paraspeckle assembly, MDA-MB-231s were transfected with ASO oligonucleotides to drive isoform switching from NEAT1_1 to NEAT1_2, resulting in a near doubling of paraspeckle numbers (Fig. 2a–c). Mirroring previous reports^14^, control MDA-MB-231s were more likely to permeate wider 10 µm channels than narrow 3 µm channels, although cells were found to be significantly faster when permeating 3 µm channels due to the well-characterized mesenchymal-to-ameboid transition (Fig. 2d, e). While paraspeckle boosting did not have an effect on permeation likelihood or velocity in narrow 3 µm channels, boosted cells were more likely to fully permeate 10 µm channels (Fig. 2f). Furthermore, boosted cells migrated significantly faster in 10 µm channels, matching the speed of control cells in 3 µm channels (Fig. 2g). Permeation through 10 µm channels was also more directional, as boosted cells were less likely to temporarily move backward during their journey (Fig. 2d).

### The paraspeckle formation is polarized in confinement

**Fig. 3:**
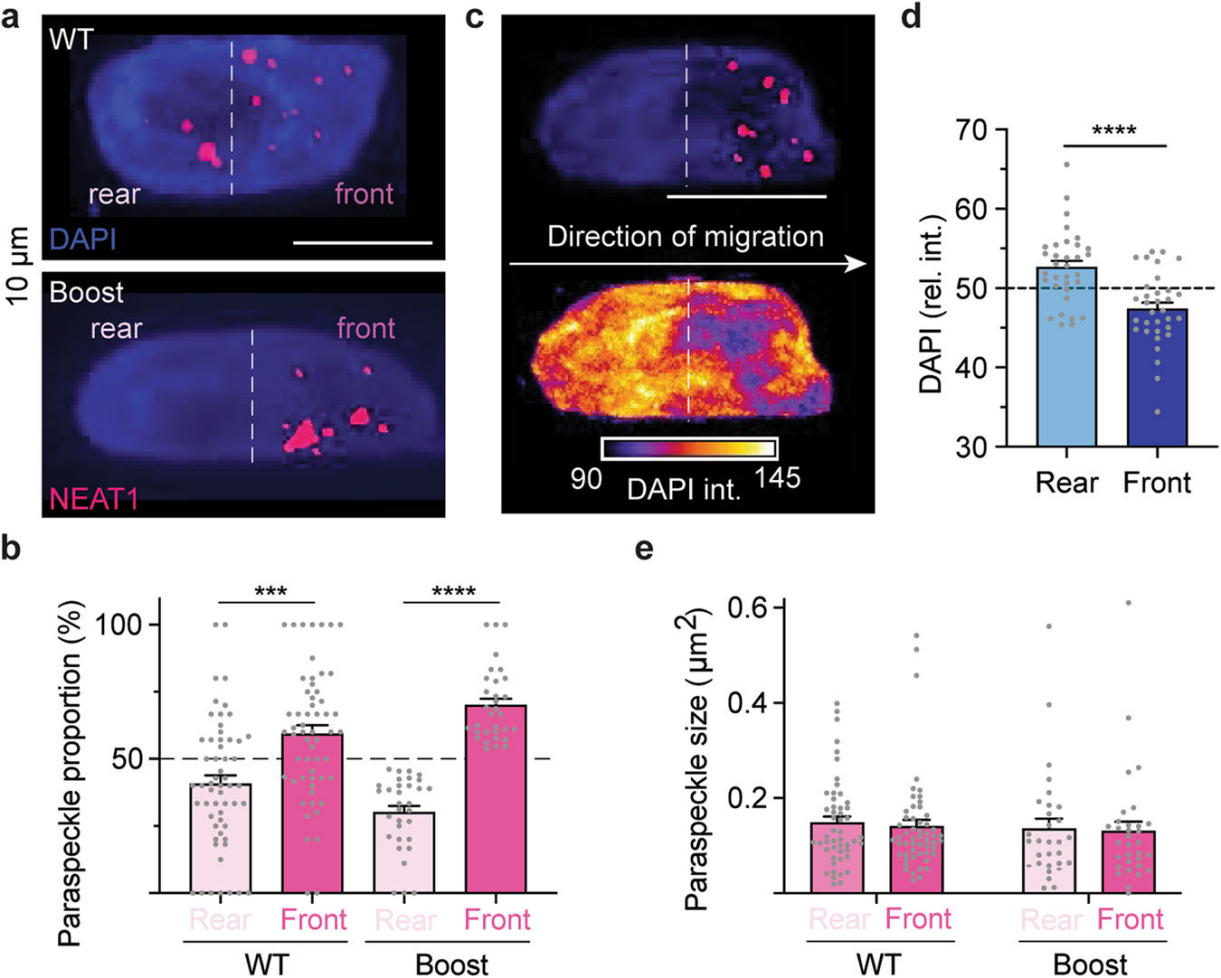
Paraspeckles are polarized in nuclei undergoing confined migration. **a** Representative RNA-FISH images of control and boost cells migrating through moderate confinement. (Scale bar, 10 µm). **b** The proportion of paraspeckles found in the front and rear of the nucleus of control and boost cells. **c** RNA-FISH image showing colocalization of polarized paraspeckles with changes in relative DAPI intensity. **d** Quantification of DAPI intensity as a function of nuclear position. **e** Paraspeckle size in control and boost cells as a function of subnuclear position showed no significant difference. All data are shown as mean ± SEM ***P < 0.001 and ****P < 0.0001. All scale bars = 10 µm.

Polarization of a number of subcellular components, including the Golgi complex^15^, the microtubule organizing center (MTOC)^16^, and membrane ion pumps^17^, have been observed during confined migration. By dividing the nucleus into front and rear halves, we found that paraspeckle formation is biased towards the leading edge of the nucleus in confinement (Fig. 3a, b). In boosted cells, paraspeckle localization was even further polarized, with over 70% of individual paraspeckles found at the leading edge of the nucleus (Fig. 3c, d). DAPI staining was then used to assess local levels of chromatin compaction^18^. In general, paraspeckles localized to regions of low DAPI intensity, indicating that paraspeckle formation is enhanced in loosely-compacted regions of euchromatin found at the front of the nucleus in confined migrating cells (Fig. 3c, d), although this did not lead to significant changes in individual paraspeckle size (Fig. 3e) and was not due to global changes in nuclear volume before, during, or after confinement (Supplementary Fig. 4). Colocalization analysis of DAPI intensity within paraspeckles revealed that in unconfined cells, paraspeckles do not materialize within less-dense chromatin regions, but in cells migrating through 10 µm confinements, paraspeckles are found in regions with significantly less DAPI signal, further suggesting that the dynamic formation of euchromatin domains in response to nuclear compression enhances phase separation of paraspeckles (Supplementary Fig. 5).

## Discussion

Recent work in the biophysics community has established that biomolecular condensates play an extraordinary role in the regulation of a wide variety of biological processes. The bulk of this research in cell models has focused on the role of biochemical perturbations on phase separation, including changes in gene expression, post-translational modification, and local protein concentration. The latter has been implicated in hyperosmotic phase separation, in which osmolarity-dependent cellular volume changes are accompanied by increased molecular crowding that is favorable for the formation of biomolecular condensates^19^. In this descriptive study, our observations here provide the first evidence of a new regime of biomolecular condensate formation to the best of our knowledge: mechanically activated phase separation (MAPS). We define MAPS condensates, such as paraspeckles, as those resulting from exposure to diverse mechanical microenvironments that directly affect local concentrations of condensate constituents. This is analogous to hyperosmotic phase separation (HOPS) condensates, which are formed as a downstream result of water efflux causing local increases in condensate constituent concentration. In contrast with HOPS condensates, the physical conditions that drive MAPS condensate formation are present in a wide variety of settings in vivo due to the fact that cells are continuously exposed to dynamic levels of compression and confinement. Here, the formation of MAPS-dependent paraspeckles is observed at the leading edge of the nucleus of cancer cells within confining microchannels. Interestingly, the favourability of MAPS formation is biphasic; in 7 and 10 µm wide microchannels, which are sufficient to increase nuclear aspect ratio by 20–50%, MAPS-induced paraspeckles nearly doubled. However, in environments that either do not compress the nucleus or those that compress it to an extreme degree, baseline paraspeckle formation is observed. The extreme compression regime corresponds with the characteristic confinements capable of inducing the mesenchymal-to-ameboid transition in cancer cells, which results in a major reorganization of the actin cytoskeleton and the nuclear lamina (Supplementary Fig. 6)^14^. Paraspeckles were also observed predominantly in regions of low chromatin compaction. This is in agreement with recent observations that liquid nuclear condensates, including nucleoli, Cajal bodies, nuclear speckles, and paraspeckles, form in areas with decreased local chromatin density^20^. In general, the findings on MAPS-induced condensates will need to be confirmed with other nuclear condensates, as well as with other types of mechanical insults such as vertical compression^21^. The role of this transient paraspeckle formation upon mechanical stimuli in phase separation mechanisms, gene expression, and cellular functions will also benefit from further investigation in the future.

Due to their association with perturbations in the biophysical environment surrounding cells, one interesting feature of MAPS class condensates is the dynamic reciprocity between condensate formation and cell behavior. We show that while confining microenvironments are sufficient to induce MAPS-induced paraspeckles, the converse is also true: driving the upregulation of paraspeckles is sufficient to enhance confined migration. We chose transient upregulation of paraspeckles via ASO as a physiologically relevant mimic of the natural increases in paraspeckles, above baseline levels, that occur in many stress scenarios. Usually, this approach would be complemented by NEAT1/paraspeckle ablation experiments. However, attempts to reduce NEAT1 via siRNA were unsuccessful in this context, due to the length of time between the delivery of siRNA and cell migration in microchannels. Hence, it remains an open question for future experiments as to whether NEAT1 loss would give an opposite phenotypic effect to the NEAT boost. Given paraspeckles are hubs of active transcription^11^, investigating changes in gene expression in paraspeckle-boosted cells, including potentially for invasion-related genes, would be a useful future experiment. Gene up-regulation may be speculated to be driven by a feed-forward enhancement of greater chromatin de-compaction as condensates form and expand within the permissive chromatin environment^20^. As cells in vivo are continuously exposed to dynamic spatiotemporal confinement, MAPS-based adaptations are likely ubiquitous across wide swaths of mechanobiology phenomena. Indeed, aquaporin-mediated water flux, which plays a key role in HOPS, has also been identified as a major effector of migration in confinement^17^. Given the rapid loss of paraspeckle production, once cells exit microchannels, we speculate that MAPS-induced paraspeckle formation upon entrance into confinement might convey a flexible migratory advantage to cancer cells in stressful microenvironments.

## Methods

### Microchannel chip fabrication

Microchannel chips were fabricated using a two-step photolithography process followed by replica molding as previously described (Supplementary Fig. 7)^14^. Polydimethylsiloxane (PDMS) (Dow Corning, Wiesbaden, Germany) with microchannel patterns were placed irreversibly onto glass coverslips. Microchannel geometries varied in widths (3, 5, 7, and 10 µm) and lengths (150 and 300 µm) while the height was consistent at 11 µm. Microchannel chips were coated with 100 µg/mL Type I Collagen in PBS (100 µg/mL) overnight at 4 °C.

### Cell culture

MDA-MB-231 human breast cancer cells (ATCC, Virginia, USA) were used throughout the study due to their well-characterized ability to invade confined spaces^14^. Cells were cultured in DMEM media (high glucose cat #11965, GibcoTM) supplemented with 10% Fetal Bovine Serum (FBS) and 1% Penicillin-Streptomycin. All experiments were performed at 37 °C in a 95% air/5% CO_2_ environment.

### Cell seeding in microchannel chips

Microchannel chips were placed in 6-well plates for FISH, while those used for live cell imaging experiments were glued into custom-cut square gaps at the bottom of a 6-well plate using Picodent dental glue (Picodent, Wipperfürth, Germany). All chips were sterilized with 305 nm UV light for 30 min prior to cell seeding. Immediately prior to seeding, 100 µL of media was added to the outer reservoirs to prevent pressure-driven flow from pushing cells into the microchannels. Cells were then seeded into the central chip reservoir at a density of 1 × 10^6^ cells/mL to ensure an optimal cell density. Cells were left to adhere for 2 h before 2 mL of media was added to each well in order to cover the chips and equilibrate inner and outer reservoir pressure. Cell-laden microchannel chips for FISH experiments were then left in the incubator for 72 h, while those used for live cell imaging experiments were transferred directly to the microscope.

### Forced NEAT1_1-NEAT1_2 isoform switching

Transfections were performed using a Neon Transfection System (Invitrogen) as per the manufacturer’s instructions with the following conditions optimized for MDA-MB-231 cells: pulse voltage (1400 V), pulse width (10 ms), and a number of pulses (4). Antisense oligonucleotides (ASOs) with a morpholino backbone were used at a concentration of 25 nM to switch the NEAT1_1 isoform to the NEAT1_2 isoform by binding to the polyadenylation site of human NEAT1 RNA. The Control ASO sequence was 5′-CCTCTTACCTCAGTTACAATTTATA and the BoostPS ASO sequence was 5′-TTTATTTGTGCTGTAAAGGG (Gene-Tools)^10^.

### Fluorescence in situ hybridization (FISH)

After 72 h of culture, cells were washed three times with PBS and fixed using 4% paraformaldehyde for 10 min, then permeabilized with 70% ethanol overnight. Paraspeckles were detected using a Stellaris RNA-FISH protocol per the manufacturer’s instructions (Stellaris, Biosearch Technologies) with a human NEAT1 5′ probe labeled with Quasar 570 dye (VSMF-2036-1; Biosearch Technologies). Nuclei were stained with 4′,6-diamidino-2-phenylindole (DAPI) (1:15,000) (Sigma-Aldrich) in DMPC-treated water for 2 min.

### Paraspeckle and DAPI analysis

All FISH-based images of paraspeckles were obtained using a DeltaVision Elite Imaging system at 60x magnification as a series of 0.2 µm-incremented Z-stacks, which were then subject to deconvolution and quick projection post-processing tasks with softWoRx software. Identical threshold parameters were applied to all images. To quantify paraspeckles, NIS-Elements Advanced Research software (Nikon) was used to segment nuclei as regions of interest (ROI) before detecting paraspeckles from binary thresholds within each ROI. To quantify paraspeckle polarization in microchannels, the ROI function was used to identify the front and rear halves of the nuclei. For DAPI polarization analysis, the nuclear area was divided into two halves by manually drawing a segmenting line perpendicular to the channels in CellProfiler. To correlate paraspeckle localization with regions of euchromatin, the intensity of the DAPI signal within paraspeckles was compared to DAPI intensity outside of paraspeckles. To quantify the total NEAT1 FISH signal for cells under confinement, or within the reservoir, a nuclear mask was defined using DAPI and total NEAT1 fluorescence within the nucleus was calculated for each image.

### Live cell imaging and analysis

Live cell imaging was performed in phase contrast using a Nikon TE300 Inverted Microscope configured with a motorized stage and on-stage incubator (OKO lab). Images were acquired from each position every 10 min over 72 h. Channel interactions were manually categorized as either penetrating (returning after partial entry to a channel), invading (returning after total entry to a channel), or permeating (moving through a channel to the opposite reservoir). The leading edges of permeating cells were tracked in Fiji using the manual tracking plugin. This data was used to calculate the average velocity (the total time to permeate divided by channel distance, averaged across all cells). In addition, live cell imaging for paraspeckle dynamics analysis was performed using MDA-MB-231 cells engineered to stably express GFP-SFPQ using a previously described method^22^. Images were obtained using a Zeiss Celldiscoverer 7 microscope and ZEN software at 37 °C and 5% CO_2_. A 13-slice Z-stack was taken at every position with a 1 µm step size every 30 min with a 50× water objective.

### Nuclear volume quantification

Nuclear volume measurements were made by staining cells for Lamin A (Abcam, ab26300) and Hoechst 33342. Images were acquired with an Olympus FV3000 NIR and a 100× oil objective, with confocal Z-stacks with a step size of 0.4 µm. The two fluorescent channels were merged using maximal pixel intensity in Fiji. Nuclear volume was then measured from the merged Z-stacks using surface creation in Imaris with local thresholding.

### Statistics and reproducibility

All data were analyzed in GraphPad Prism (9.0). Unpaired one-way ANOVA with Kruskal–Wallis test and unpaired student’s *t*-test with Mann–Whitney test were performed. All other data were analyzed using a student’s *t*-test. All experiments were repeated at least three times. Results are presented as mean ± SEM.

### Reporting summary

Further information on research design is available in the Nature Portfolio Reporting Summary linked to this article.

## Supplementary information

Supplementary Information

Description of Additional Supplementary Files

Supplementary Data 1

Supplementary Video 1

Reporting Summary

## Data Availability

Source data are provided in Supplementary Information.
