# Supplementary material for: Confined environments induce polarized paraspeckle condensates
Source: Commun Biol. 2023 Feb 3;6:145. doi: 10.1038/s42003-023-04528-4 (PMC9898560; doi:10.1038/s42003-023-04528-4)
Supplement: Supplementary file 1 — Supplementary Information [file 42003_2023_4528_MOESM1_ESM.pdf]

Supplementary Information

Confined environments induce polarized paraspeckle condensates

Vanja Todorovski[a], Finn McCluggage[a], Yixuan Li [b], Annika Meid[c,d], Joachim P. Spatz[c,d], Andrew W. Holle[b,e,1], Archa H. Fox[a,1], Yu Suk Choi[a,1]

[a] School of Human Sciences, The University of Western Australia, Crawley 6009, WA, Australia
[b] Mechanobiology Institute, National University of Singapore, 117411, Singapore
[c] Department of Cellular Biophysics, Max Planck Institute for Medical Research, Heidelberg 69120, Germany
[d] Department of Biophysical Chemistry, University of Heidelberg, Heidelberg 69117, Germany
[e] Department of Biomedical Engineering, National University of Singapore, 117411, Singapore

[1] Corresponding authors
To whom correspondence should be addressed.
E-mail: bieawh@nus.edu.sg, archa.fox@uwa.edu.au, or yusuk.choi@uwa.edu.au

Supplementary Figures

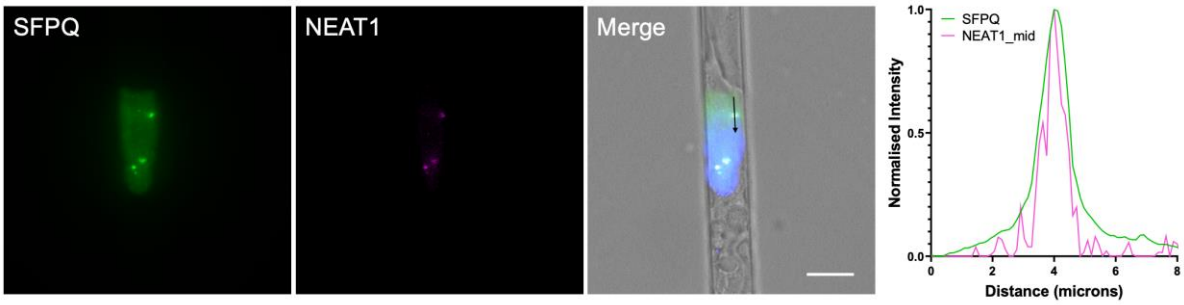

Supplementary Figure 1. NEAT1 FISH and SFPQ immunofluorescence colocalization. All scale bars = 10 µm.

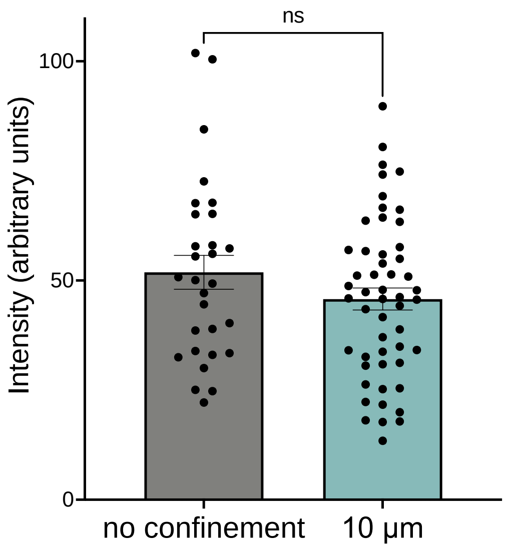

Supplementary Figure 2. NEAT1 FISH intensity under no confinement and 10 µm confinement conditions. All data shown as mean ± SEM.

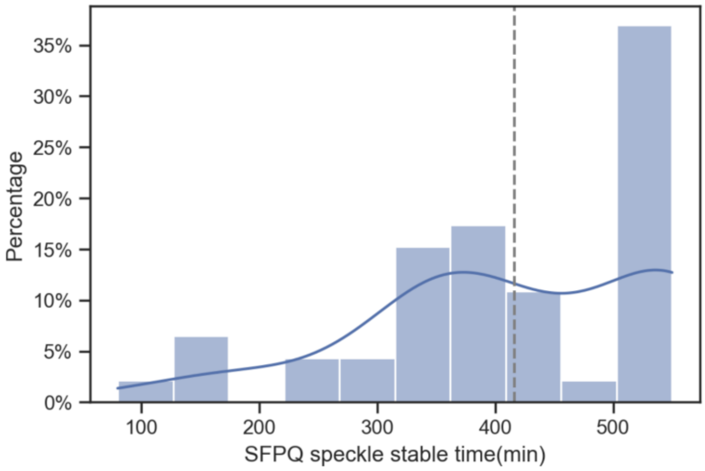

Supplementary Figure 3. Paraspeckle stability measured as a function of time. The majority of paraspeckle condensates are stable for periods of time longer than 5 hours, which is longer than the time cells spend in confinement.

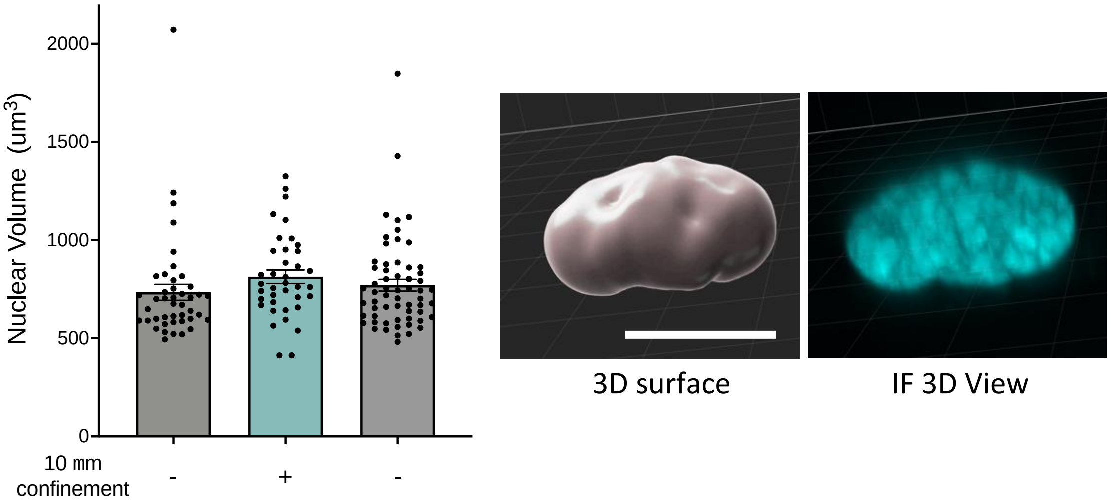

Supplementary Figure 4. Nuclear volume before, during, and after 10 µm confinement. All data shown as mean ± SEM. One-way ANOVA showed no differences. All scale bars = 10 µm.

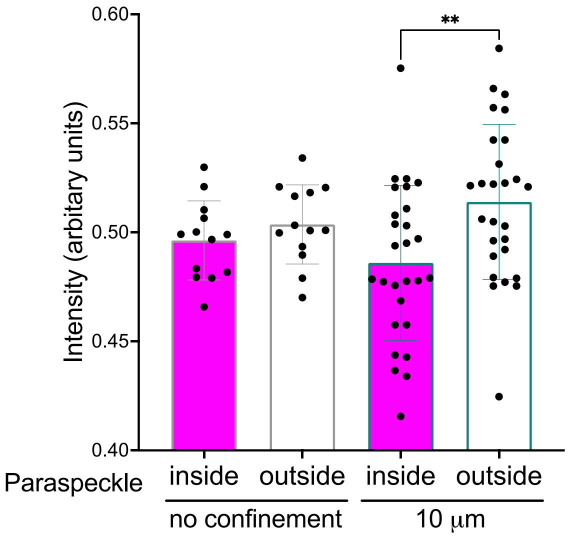

Supplementary Figure 5. DAPI intensity inside and outside of paraspeckles. All data shown as mean ± SEM. One-way ANOVA showed significance only in inside vs. outside in 10 μm confinement. ** p=0.0089

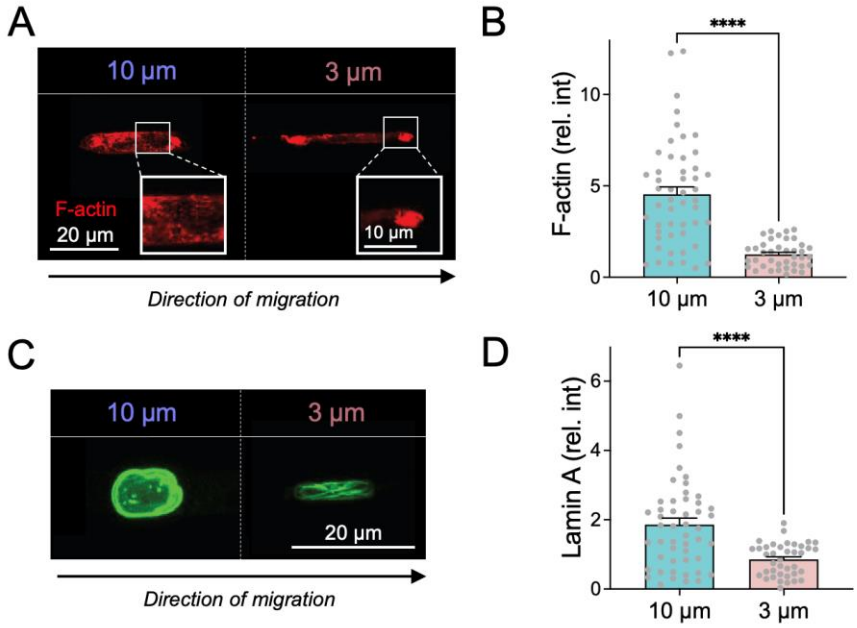

Supplementary Figure 6. Intensities of F-actin and Lamin-A in cells under 10 μm and 3 μm confinement. All data shown as mean ± SEM. Student's t-test with Mann-Whitney test showed significance 10 μm vs. 3 μm. **** p<0.0001.

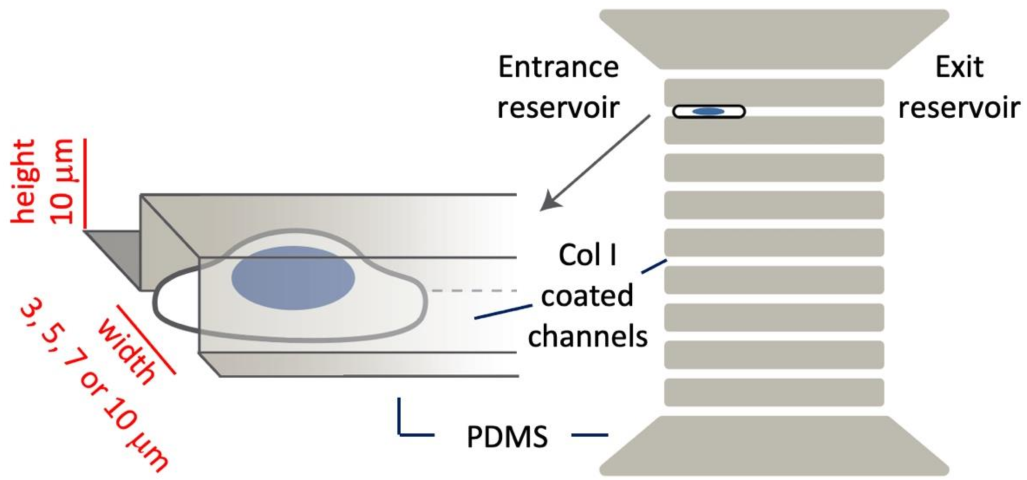

Supplementary Figure 7. Schematic drawing of microchannel chips.
