# Supplementary material for: Confined environments induce polarized paraspeckle condensates
Source: Commun Biol. 2023 Feb 3;6:145. doi: 10.1038/s42003-023-04528-4 (PMC9898560; doi:10.1038/s42003-023-04528-4)
Supplement: Supplementary file 2 — Description of Additional Supplementary Files [file 42003_2023_4528_MOESM2_ESM.pdf]

Description of Additional Supplementary Files

File name: Supplementary Data 1

Description: Raw data for all graphs, figures 1b, 1d, 2b, 2c, 2f, 2g, 3b, 3d, and 3e and supplementary figures 2, 4, 5, 6b and 6d, are presented in the Excel spreadsheet.

File name: Supplementary Video 1.

Description: Time lapse video of paraspeckle changes before, during and after confinement labelled by GFP-SFPQ.
